# Recognition of Emotional and Neutral Visual Scenes in Carriers of the MAOA, COMT, DRD4, and 5HT2A Gene Polymorphisms

**DOI:** 10.11621/pir.2022.0410

**Published:** 2022-12-30

**Authors:** Pavel N. Ermakov, Elena V. Vorobyeva, Ekaterina G. Denisova, Denis V. Yavna, Vitali V. Babenko, Ekaterina M. Kovsh, Daria S. Alekseeva

**Affiliations:** a Southern Federal University, Rostov-on-Don, Russia; b Don State Technical University, Rostov-on-Don, Russia

**Keywords:** Visual scene recognition, MAOA, COMT, DRD4, 5HT2A genes

## Abstract

**Background:**

It is known that some genes regulate neurochemical metabolism, and their polymorphisms affect cognitive performance, including the ability to categorize emotionally significant information.

**Objective:**

The aim of our study was to analyze the recognition of emotional and neutral visual scenes in carriers of different polymorphic variants of the MAOA, COMT, DRD4, and 5HT2A genes.

**Design:**

The study sample consisted of 87 university students (Caucasians, women 63%, average age 20.4±2.6 years). The genotypes of the COMT, 5HT2A, and DRD4 genes were determined by polymerase chain reaction. Agarose gel electrophoresis was used to determine the number of tandem repeats of the MAOA gene. Three hundred sixty (360) photographic images of scenes of different emotional valence (positive, negative, and neutral — 120 images for each category) were used as stimuli. These images were classified by expert assessments. The images were presented in a random sequence. The exposure time was 700 ms. The research participants were asked to determine the emotional valence of each scene.

**Results:**

We found that only the COMT gene genotype affected the recognition of emotional and neutral visual scenes. Carriers of the COMT Val/Val genotype, which causes dopamine to stay in the synaptic space for a shorter time, are better in recognizing and demonstrate higher sensitivity to the emotional content of scenes. Carriers of the Val/Met genotype demonstrated the worst ability to differentiate the emotional valence of visual scenes.

**Conclusion:**

This study has shown that the length of stay of monoamines in the synaptic space regulated by the COMT gene affects the recognition of emotional visual information.

## Introduction

Visual scene recognition is one of the fundamental operations people use to build an internal model of the world. One of the important semantic characteristics of visual scenes is their emotional valence (You & Li, 2016). The individual features of recognition of emotional scenes affect cognitive processes and are an important personal psychological characteristic (Bush et al., 2015). When a person’s ability to recognize emotions is impaired (a condition called alexithymia), his/her ability to perceive negative visual scenes worsens (Wang et al., 2021).

In a study using EEG and fMRI simultaneously while the subjects were viewing visual scenes of pleasant, unpleasant, and neutral content, it was found that affective scenes, depending on their valence, evoked specific neural representations (Bo et al., 2021). The influence of the scene’s emotionality on the neuronal response was also shown using evoked potentials (Tebbe et al., 2021; Trujillo et al., 2021). There was also evidence of valence-modulated susceptibility to the image distortions when observing the scenes (Stevenson et al., 2020).

Emotional scene recognition has both universal patterns and individual characteristics, which are determined by the person’s genotype. In a study on 233 pairs of twins at the age of 11 years, it was discovered that the children’s gaze trajectory during the perception of visual scenes was significantly influenced by genetic factors (Kennedy et al., 2015).

Prior study has demonstrated that recognizing emotionally significant information is greatly influenced by the activity of selective serotonin and noradrenaline reuptake inhibitors (Hamrer, 2012). The organization of these synaptic mechanisms is known to be largely genetically predetermined. In particular, the deletion of the ADRA2B gene that encodes the a2b-adrenergic receptor has been shown to increase reactivity to emotional stimuli (Li, 2013) and affect emotional memory (Maheu et al., 2004; de Quervain et al., 2007;). However, the more obvious candidates for genes whose polymorphisms affect the perception of emotionally significant information are those of the dopaminergic (DRD4, COMT) and serotonergic (MAOA, HTR2A) systems (Ermakov et al., 2017; Vorobyeva et al., 2020).

The monoamine oxidase A (MAOA) gene is located on the X chromosome and regulates the duration of the dopamine, serotonin, and norepinephrine stay in the synaptic cleft. . Carriers of the MAOA gene alleles with two, three, and five repeats in the promoter region have a low-active form of the gene and enzyme that catabolizes neurotransmitters, which leads to a longer stay of dopamine, norepinephrine, and serotonin in the synaptic cleft ; carriers of alleles with 3.5, 4, and 3.5/4 repeats have a highly active monoamine oxidase A, which leads to a shorter stay of monoamines in the synaptic cleft (Brady, 2012). Low- and high-activity genotypes for the MAOA-uVNTR are associated with the functioning of the mesolimbic subsystem of the dopamine system, cingulate gyrus, amygdala, nucleus accumbens, hippocampus, and prefrontal cortex. There is also evidence that MAOA-uVNTR gene polymorphism is indirectly associated with the personal traits and processing of stimuli of various valences (Barnett et al., 2011; Williams et al., 2009).

The COMT gene is located on chromosome 22. It encodes the production of the Catechol-O-methyltransferase enzyme, which plays an important role in the breakdown of catecholamines (dopamine, adrenaline, and norepinephrine). The single nucleotide polymorphism rs4860 (Val158Met) can be represented by the G allele encoding the amino acid valine, or the low active A allele encoding the amino acid methionine. Carriers of the A (Met) allele have an increased length of stay of the dopamine in the prefrontal cortex; they also have a higher pain threshold, lower stress resistance, and more efficient cognitive function. Carriers of the G (Val) allele, on the other hand, have decreased length of stay of catecholamines in the synaptic space due to the high activity of the COMT enzyme, as well as an increased sensitivity to pain, higher stress resistance, and a moderate decline in the prefrontal function (Chen et al., 2004; Lotta et al., 1995).

It has been shown that the Val158Met single nucleotide polymorphism of the COMT gene affects individual differences in attention, while insufficient or excessive catecholaminergic activity can each have equally undesirable consequences for attention span (Shalev et al., 2019). Serrano et al. have described the psychological characteristics (mainly related to the stress response) of carriers of different COMT genotypes (Serrano et al., 2019). It has been shown in a Russian sample that the presence of the Val/Val genotype in the COMT gene is associated with a high risk of depression (Gafarov et al., 2021).

The DRD4 gene is located on chromosome 11. It encodes the D4 subtype of the dopamine receptor. The dopamine D4 receptor is involved in transmitting neural signals to the brain’s mesolimbic system, which regulates emotions and complex behavior (Wang et al., 2017). The association of single nucleotide polymorphisms (SNPs) of the DRD4 gene with the functioning of working memory and perception was shown in a study on a South American family-based sample (Cervantes-Henriquez et al., 2021).

The HTR2A gene that encodes the 5-HT2A receptor (5-hydroxytryptamine (serotonin) receptor 2A) is localized on the 13q14-q21 chromosome. This gene is involved in the serotonin system and is oft en of interest to scientists who have been searching for genetic associations of various psychopathologies such as schizophrenia (González-Castro et al., 2013; Mistry et al., 2011; Yan et al., 2021), but the features of its relationship with emotional and cognitive processes in healthy people have not been sufficiently studied.

Based on the above, we assumed that the described genes could be considered factors associated with human emotional reactions. However, in previous years, the relationship of these and a number of other genes with the perception of emotional scenes was studied mainly in connection with the development of pathological processes: affective disorders (Cipriani et al., 2009) or Alzheimer’s disease (Cavill, 2017).

The aim of our study was to study the features of recognition of emotionally colored and neutral visual scenes in healthy carriers of the different genotypes of the MAOA, COMT, DRD4, and 5HT2A (HTR2A) genes.

## Methods

### Participants

Our study involved 87 university students (Caucasians, women 63%) ages 19 to 25 years (average age 20.4 ± 2.6). All participants had normal or normalized vision, and no history of neurological or psychiatric illness. At the initial stage of the process, all participants were informed about the research objectives and procedures, and gave written consent for voluntary participation.

### Procedure

#### Stimuli

Three hundred sixty (360) photographic images of scenes of different emotional valence (positive, negative, and neutral — 120 images for each category) were used as stimuli. The images were obtained from open sources and classified as positive, negative, or neutral by independent experts. Selected scenes are freely available now as a dataset at https://osf.io/eagyc/. We aligned the images by average brightness and RMS contrast. The image width was 22.5, and the height was 18 angular degrees.

#### Procedure

Participants were positioned in a head-chin rest at 60 cm distance from the center of the screen. The images of the scenes were presented in a random sequence. The exposure time was 700 ms. Prior to the experiment, all subjects underwent brief training that helped familiarize them with the procedure and ensured that they understood the task correctly. The training images did not appear in the main experiment. The duration of the main experiment did not exceed 25 minutes, and the experimental task was not tiring. The subjects were tasked to recognize the emotional valence of each scene shown and report their decision by clicking one of the three response options (positive, negative, or neutral). The demonstration of the next stimulus was launched 100 ms after the participant’s response.

#### Genotyping

Genetic analysis was carried out in the “Biological Solutions and Technologies” laboratory (Russian Federation, Moscow). DNA was extracted from buccal cells. To determine the genotypes of the COMT, 5HT2A, and DRD4 genes, the genotyping procedure was carried out by using the real-time PCR method (Real-Time CFX96 Touch cycler, Bio-Rad, USA). An agarose gel electrophoresis procedure was used to determine the number of MAOA tandem repeats.

#### Statistical data analysis

The number of correct recognitions and “false alarms” for each type of image was determined. Next, the sensitivity index (d’) was calculated using the PAL_SDT_1AFC_ PHFtoDP function from the Palamedes toolbox (Prins & Kingdom, 2018). The probability of correctly identifying an emotion (hits) was considered as pH, and the probability of false alarms (fails) was considered as pF. For example, for a neutral image, pH is equal to the ratio of the number of “neutral” responses to stimuli expressing neutral scenes, to their total number; pF is the ratio of the number of “neutral” responses to positive and negative scenes, to the total number of such stimuli. The calculation comes down to subtracting the normalized pF from the pH for each type of stimulus (Prins & Kingdom, 2018). The overall (general) sensitivity index was calculated as the average d’ for the three types of scenes.

For intergroup comparisons of index sensitivity in carriers of different genotypes of the studied genes, we used the Kruskal-Wallis Test. As a post-hoc procedure, Dunn’s test with Holm’s correction for multiple comparisons was used. Statistical data processing was carried out using an open-sourced JASP Computer soft ware (Version 0.16, 2021).

## Results

We have analyzed the following DNA sections: the Val158Met polymorphic locus of the COMT gene (472A>G, rs4680), tandem repeats (VNTR) in the promoter region of the MAOA gene, the HTR2A gene for the 5HT2A serotonin receptor (T102C rs6313, Tr3) and the DRD4 gene for D4 subtype dopamine receptor (\-C-521T, rs1800955).

The following genotypes were found in our sample for the MAOA gene: highly active H genotypes, including 4 tandem repeats (VNTR) of the promoter region of the gene; low-active L genotypes with 5 tandem repeats (VNTR) of the promoter region of the gene; and heterozygous medium active genotypes M, including alleles with 5 and 4 tandem repeats (VNTR) of the promoter region of the gene. The distribution of genotype frequencies of the studied genes is presented in *Table 1* and corresponds to the Hardy-Weinberg equilibrium.).

**Table 1:** Frequency distribution of MAOA, COMT, DRD4, and 5HT2A gene genotypes in the study sample

Gene polymorphisms	Genotypes and their distribution in the sample (in percents)		
Gene MAOA (VNTR)	H genotypes (4 repeats of the promoter region of the gene)	L genotypes (5 repeats of the promoter region of the gene)	M genotypes (5/4 repeats of the promoter region of the gene)
	22%	50%	28%
Gene COMT (Val158Met, rs4680)	Val/Val	Met/Met	Val/Met
	16%	29%	55%
Gene DRD4 (C-521T, rs1800955)	T/T	C/T	C/C
	39%	50%	11%
Gene 5HT2A (T102C rs6313, Tr3)	C/C	C/T	T/T
	34%	50%	16%

We proceeded to the main part of the study after processing the results of the genetic analysis. *Table 2* shows the differences in the accuracy of recognition of the emotional content of visual scenes in carriers of different genotypes of the MAOA, COMT, DRD4, and 5HT2A genes.

**Table 2:** The results of the Kruskal-Wallis Test for comparing sensitivity to the emotional valence of visual scenes in carriers of different genotypes of the MAOA, COMT, DRD4, and 5HT2A genes (* - p<0,05)

Genes and genotypes		General sensitivity index	Visual scenes		
			Negative	Neutral	Positive
Mean ranks for COMT (Val158Met, rs4680)	Val/Met	38.52	40.29	37.37	39.75
	Met/Met	45.80	43.98	48.22	44.72
	Val/Val	60.91	57.00	60.64	57.77
Kruskal-Wallis test	H	7.548	4.051	9.088	4.782
	p	0.023_*_	0.132	0.011_*_	0.092
Mean ranks for 5HT2A (T102C rs6313, Tr3)	C/T	46.67	48.01	44.51	43.42
	C/C	41.34	38.79	42.86	44.90
	T/T	36.83	37.96	41.25	40.42
Kruskal-Wallis test	H	1.795	3.091	0.190	0.274
	p	0.408	0.213	0.909	0.872
Mean ranks for DRD4 (C-521T, rs1800955)	C/T	40.38	41.28	41.01	42.68
	T/T	45.06	43.81	46.89	46.56
	C/C	48.27	48.60	42.73	39.10
Kruskal-Wallis test	H	1.279	0.939	0.991	0.989
	p	0.528	0.625	0,609	0.610
Mean ranks for MAOA (VNTR)	H	37.78	36.13	38.80	37.07
	L	44.83	43.38	45.87	47.37
	M	45.14	49.79	42.00	40.98
Kruskal-Wallis test	H	1.411	3.379	1.252	2.754
	p	0.494	0.185	0.534	0.252

*Note. Asterisks indicate statistically significant results*.

**Figure 1.**
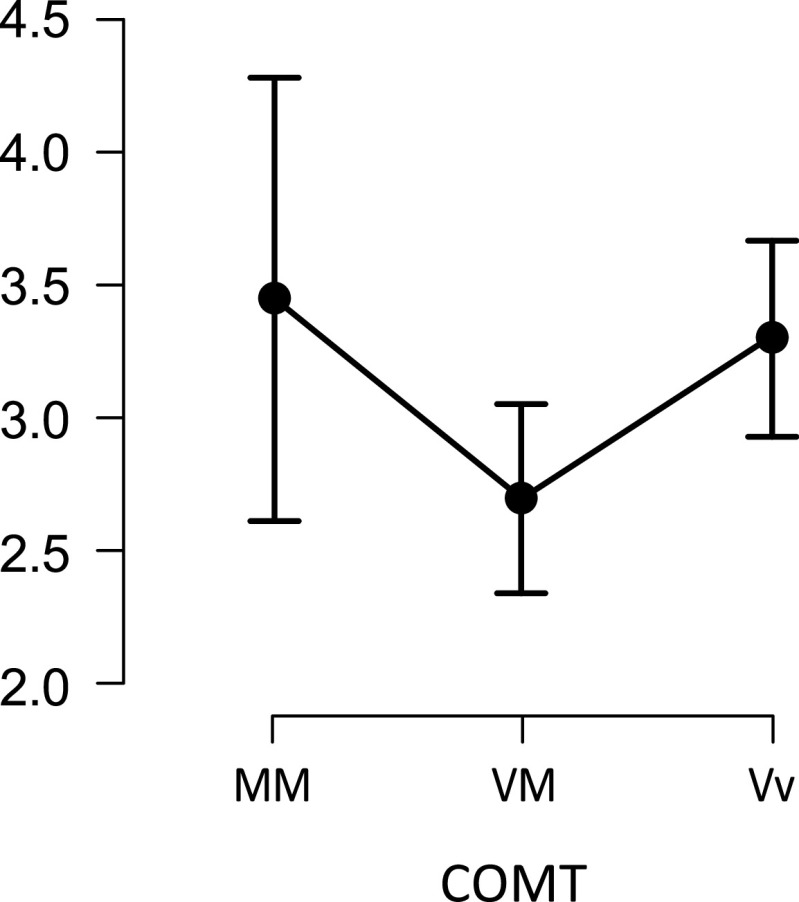
Accuracy of recognition of neutral scenes by carriers of different genotypes of the COMT gene.

*Note*. Y-axis — sensitivity (d’). The abscissa shows genotypes for the COMT gene: MM (Met/Met), VM (Val/Met), and VV (Val/Val).

The Kruskal-Wallis Test showed significant differences in sensitivity to the neutral scenes in carriers of different COMT gene genotypes (H = 9.1, df = 2, p = 0.01). The Dunn’s Post Hoc Comparisons showed that carriers of the heterozygous genotype were less successful at recognizing neutral images (*Figure 1*). At the same time, the recognition accuracy of neutral scenes in Val/Met was statistically significantly lower than in Val/Val (VM — VV, z = –2.798, _pholm_ = 0.008).

**Figure 2.**
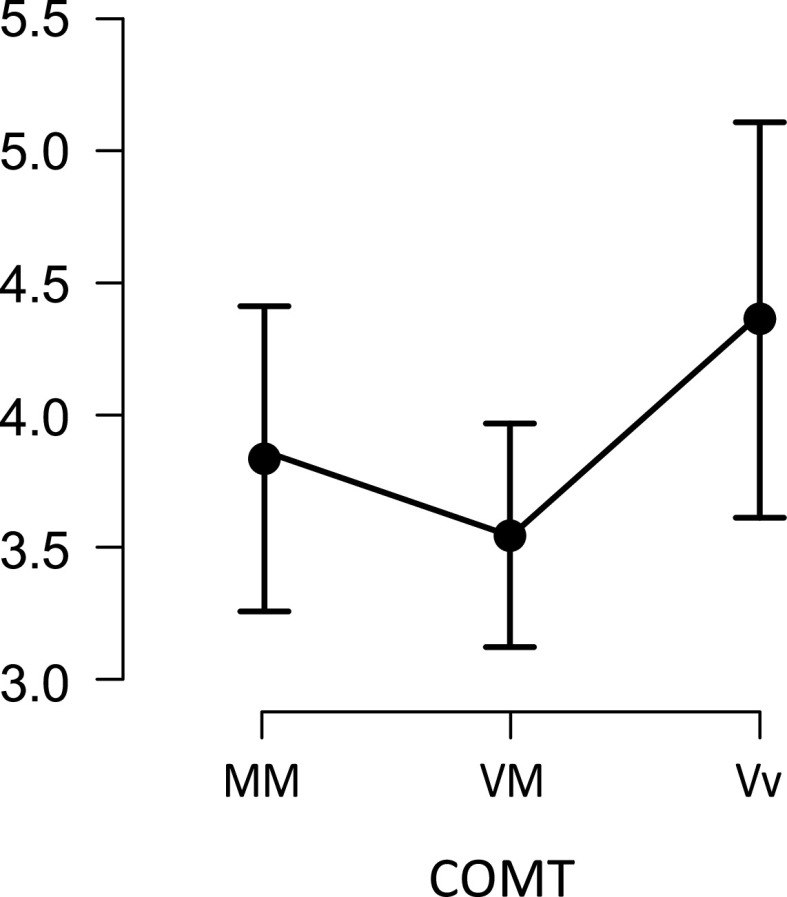
Accuracy of recognition of the emotional valence of scenes by carriers of different genotypes of the COMT gene.

*Note. Y-axis — sensitivity (d’). The abscissa shows genotypes for the COMT gene: MM (Met/Met), VM (Val/Met), and VV (Val/Val)*.

The Kruskal-Wallis Test showed significant differences in sensitivity to the emotional valence of scenes by carriers of different COMT gene genotypes (H = 7.458, df = 2, p = 0.02). Dunn’s Post Hoc Comparisons showed that carriers of the Val/Val genotype were generally better than others in recognizing the emotional valence of stimuli (*Figure 2*). At the same time, the accuracy of recognition of the emotional valence of scenes in Val/Val was statistically significantly higher than in Val/Met (VM – VV, z = –2.692, _pholm_ = 0.011).

## Discussion

Our results show that of the four genes studied, only COMT polymorphism was associated with the perception of emotional visual scenes. Moreover, statistically significant differences were obtained only when comparing the Val/Val and Val/Met groups. It turned out, in particular, that carriers of the Val/Met genotype for the COMT gene were worse in recognizing neutral and emotional scenes than carriers of the Val/Val genotype.

Our finding is consistent with the results of a previous meta-analysis, which showed an association of the Met allele with reduced emotional processing efficiency (Mier et al., 2010). A more recent study discovered that Val/Met heterozygotes retain fewer details of past emotional events than carriers of other genotypes (Tõugu et al., 2021). What can explain it? It is possible that the length of stay of monoamines in the synaptic space, which is regulated by the COMT gene, is causing those differences. A number of authors have posited that carriers of the Val/Met COMT genotype have a longer stay of monoamines in the synaptic space compared to carriers of the Val/Val genotype (Chen et al., 2004; Lotta et al., 1995). Our results indicate that a longer stay of monoamines in the synaptic space is a less preferable factor in tasks for recognizing the emotional valence of visual scenes.

The decline in scene categorization performance in Val/Met carriers may also be associated with increased levels of dopamine in the prefrontal cortex. Analysis of the data presented by Zareyan et al. (2021) indicates that the presence of the Met allele at codon 158 results in higher levels of dopamine in this cortical region than the presence of the Val allele does. It is possible that successful recognition of neutral and emotional visual scenes does not require an increased amount of dopamine in the prefrontal cortex. On the contrary, its increased levels worsen the performance.

Thus, the greater sensitivity of carriers of the Val/Val COMT genotype to the emotional content of visual scenes may be associated with a faster breakdown of monoamines (including dopamine) in the synaptic space, as well as with a relatively low level of dopamine in the prefrontal cortex.

## Conclusion

This study, carried out on healthy subjects, made it possible to obtain new data concerning the mechanisms of recognition of emotional visual information and the influence of genes involved in the regulation of neurochemical metabolism on these processes. Our results specify the role of the COMT gene polymorphism in the perception of emotionally significant signals and allow us to draw the following conclusions:

1. Among the examined genes (MAOA, COMT, DRD4, and 5HT2A), only the COMT gene genotype affects the recognition of emotional and neutral visual scenes.
2. Carriers of the COMT Val/Val genotype, which causes dopamine to stay in the synaptic cleft for a shorter time, recognize neutral scenes better and demonstrate a higher sensitivity to the emotional content of scenes.
3. Carriers of the COMT Val/Met genotype are less successful at recognizing the emotional valence of visual scenes.

## Limitations

The limitation of our study is related to the unequal number of persons per genotype in the sample. However, such unevenness of genotypes occurs naturally in the human population.
